# Fine‐scale population differences in Atlantic cod reproductive success: A potential mechanism for ecological speciation in a marine fish

**DOI:** 10.1002/ece3.4615

**Published:** 2018-10-31

**Authors:** Nancy E. Roney, Rebekah A. Oomen, Halvor Knutsen, Esben M. Olsen, Jeffrey A. Hutchings

**Affiliations:** ^1^ Department of Biology Dalhousie University Halifax Nova Scotia Canada; ^2^ Centre for Ecological and Evolutionary Synthesis (CEES), Department of Biosciences University of Oslo Oslo Norway; ^3^ Institute of Marine Research Flødevigen Marine Research Station His Norway; ^4^ Centre for Coastal Research (CCR) University of Agder Kristiansand Norway

**Keywords:** Atlantic cod, broadcast spawning, fjord, *Gadus morhua*, mating, parentage

## Abstract

Successful resource‐management and conservation outcomes ideally depend on matching the spatial scales of population demography, local adaptation, and threat mitigation. For marine fish with high dispersal capabilities, this remains a fundamental challenge. Based on daily parentage assignments of more than 4,000 offspring, we document fine‐scaled temporal differences in individual reproductive success for two spatially adjacent (<10 km) populations of a broadcast‐spawning marine fish. Distinguished by differences in genetics and life history, Atlantic cod (*Gadus morhua*) from inner‐ and outer‐fjord populations were allowed to compete for mating and reproductive opportunities. After accounting for phenotypic variability in several traits, reproductive success of outer‐fjord cod was significantly lower than that of inner‐fjord cod. This finding, given that genomically different cod ecotypes inhabit inner‐ and outer‐fjord waters, raises the intriguing hypothesis that the populations might be diverging because of ecological speciation. Individual reproductive success, skewed within both sexes (more so among males), was positively affected by body size, which also influenced the timing of reproduction, larger individuals spawning later among females but earlier among males. Our work suggests that spatial mismatches between management and biological units exist in marine fishes and that studies of reproductive interactions between putative populations or ecotypes can provide an informative basis on which determination of the scale of local adaptation can be ascertained.

## 1 INTRODUCTION

The persistence of a species depends on the number, phenotypic differentiation, and genetic variability of its constituent populations (Schindler et al., 2010). Population identification in most marine fishes, however, remains a fundamental challenge, in part because of potentially high mobility. The ocean environment tends to facilitate dispersal at one or more developmental stages, resulting in species distributions that span broad expanses. Additional challenges stem from observations that population connectivity is generally higher in the marine realm than it is for terrestrial systems (Hauser & Carvalho, 2008) such that even limited genetic exchange between populations can erode genetic divergence. Fishery and conservation management units are often established at very large spatial scales (e.g., COSEWIC 2010), although many appear to have limited conformity with independent demographic units and are often based on decades‐old interpretations of the flow patterns of ocean currents (Cadrin, Friedland, & Waldman, 2004).

The Atlantic cod (*Gadus morhua*) is a prime example of such a species. Management units in the Northwest Atlantic have remained unchanged since the 1940s (ICNAF, 1952), the largest extending more than 1,000 km, despite data indicating that some of these large units contain multiple, smaller units that represent biologically and genetically distinct populations. For example, the Southern Scotian Shelf/Bay of Fundy fisheries management unit (Northwest Atlantic Fishery Organization Division 4X) includes groups of cod that have spatially distinct spawning locations, temporally different spawning periods, and genetically different responses to temperature change (Hutchings et al., 2007; Oomen & Hutchings, 2015, 2016 ).

Similar mismatches between the spatial scales of management and putative local adaptation almost certainly exist in the Northeast Atlantic. All cod that inhabit Norwegian coastal waters north of 62° are managed as a single unit ( www.ices.dk), despite evidence of variation in genetic structure, spawning location, and spawning time within the unit (Johansen et al., 2009). South of 62°, Norwegian cod are considered part of a single North Sea cod management unit ( www.ices.dk). Yet cod that inhabit fjords and coastal waters along the southeast Norwegian coast—Skagerrak—can differ genetically from North Sea cod (Knutsen et al., 2018, 2011 ), a distinction that seems temporally stable, suggesting that cod spawn in, and inhabit, coastal Skagerrak waters throughout the year rather than the North Sea (Cianelli et al., 2010; Knutsen et al., 2007, 2011 ; Rogers et al., 2011).

There is compelling evidence that cod inhabiting the inner waters of some Skagerrak fjords are phenotypically and genetically distinct from cod inhabiting outer waters of the same fjord (Knutsen et al., 2018; Øresland & André, 2008). In this respect, the most extensive data are available for Risør Fjord (58.7°N, 9.2°E). Although they are not physically restricted from moving between the two areas, cod inhabiting inner and outer Risør waters can differ genetically from one another (Knutsen et al., 2011). Recent analyses of single nucleotide polymorphism (SNP) data have revealed that two different genotype clusters of cod coexist in various proportions along coastal Skagerrak (Knutsen et al., 2018); a “fjord” ecotype dominates the waters of the inner fjords, for which Risør is an excellent example, whereas a “North Sea” ecotype is often predominant in outer‐fjord waters. Phenotypically, cod from inner Risør grow at a slower rate than those from outer Risør (Kuparinen, Roney, Oomen, Hutchings, & Olsen, 2016). Differences in life‐history traits are evident among several coastal Skagerrak fjords and appear to be spatio‐temporally stable (Roney et al., 2016).

Thus, Risør Fjord would appear to be an ideal location in which to study questions related to the spatial scale of local adaptation in marine fish with high dispersal capabilities. However, notwithstanding the phenotypic and genetic differences between the two populations described above, it is not known whether individuals would have equal reproductive success in a mixed‐population spawning situation. Here, we explore this hypothesis. Equivalence in reproductive success under such conditions would imply an absence of intrinsic barriers to interbreeding attributable to potential differences in factors likely to affect mating probability, such as body size, behavior, physiology, and (or) gamete quality. In contrast, if one population experiences higher average reproductive success when cohabiting the same breeding environment, ceteris paribus it might indicate that genetic and life‐history differences between inner and outer populations reflect processes contributing to reduced probability of interbreeding, and increased probability of reproductive isolation, between populations (Rundle & Nosil, 2005; Schluter, 2000).

## 2 MATERIALS AND METHODS

### 2.1 Parental fish collection

**Figure 1:**
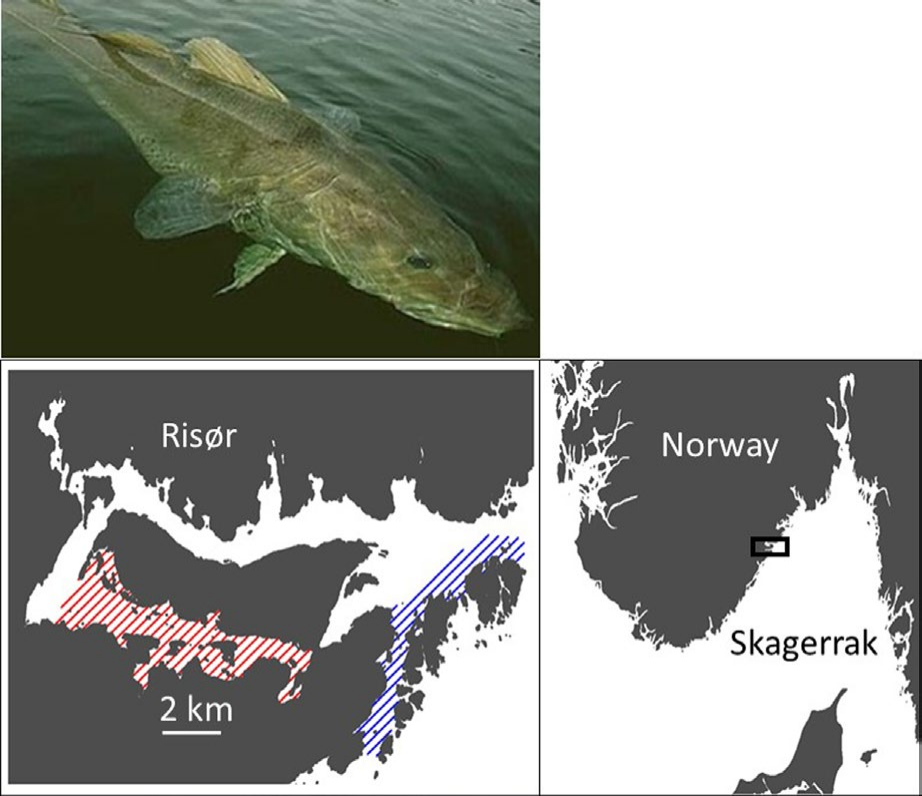
Atlantic cod were collected from the inner (red‐lined area) and the outer (blue‐lined area) Risør Fjord on the Norwegian Skagerrak coast. Modified from Kuparinen et al. (2016)

Skagerrak (Figure 1) is a strait bounded by southeast Norway, southwest Sweden, and Denmark's Jutland peninsula, connecting the North Sea and the Kattegat sea area (the latter being the entrance to the Baltic Sea). The Norwegian Skagerrak is highly heterogeneous, comprising a multitude of habitats ranging from sheltered mud flats and wave‐exposed cliffs to semi‐enclosed fjords and deep (~700 m) near‐coast waters (Sætre, 2007).

Risør Fjord (Norwegian Skagerrak) encompasses ~20 km^2^, providing habitat for two putative populations of cod, one inhabiting the inner fjord and the other the outer fjord. Four to six weeks prior to spawning (December 2014), adults were collected by fyke net from the inner and outer fjord at Sørfjorden and Østerfjorden, respectively. Fish were measured for length, tagged externally (using a T‐Bar anchor tag labeled with a unique identification code), and subsequently placed in a single spawning basin at the Institute of Marine Research Flødevigen Research Station (~60 km south of Risør) where they spawned undisturbed in a ~1‐m deep, 9 m × 5 m spawning basin lined with natural rock. Although the overall sex ratio (determined by postmortem inspection) was female‐biased 1.6:1.0 (45 females, 28 males), sex ratio did not differ between populations (χ^2^ = 0.40, *p* = 0.53), the number of females:males being 24:12 and 21:16 for outer‐fjord (length range: 45–63 cm) and inner‐fjord cod (45–57 cm), respectively. Water in the spawning basin, pumped regularly from a depth of 75 m, averaged 7.4°C (ambient temperature). Lights were adjusted to mimic the natural photoperiod and cod were fed ~2 kg of frozen shrimp daily.

### 2.2 Offspring sampling protocol

**Figure 2:**
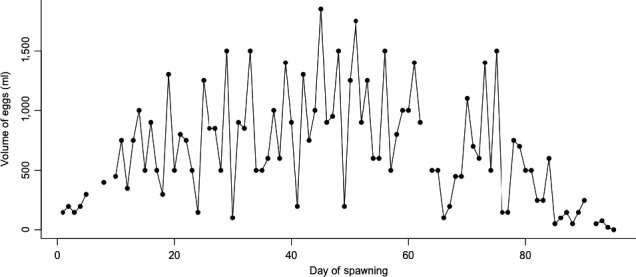
Daily volume (ml) of eggs collected throughout the spawning period (20 January–24 April 2015). Absent estimates of egg volume were due either to an absence of eggs or an overflow of the egg collector, resulting in an inaccurate egg‐volume estimate

Eggs were sampled daily (between 08:00 and 10:00 hours), using a small container through which the spawning‐basin outflow passed. The spawning period began when eggs were first evident in the egg collector (20 January 2015) and ended when no eggs had been collected for five consecutive days (the final date of egg collection was 24 April 2015; Figure 2). Eggs were collected on 90 of the 94 days that comprised the spawning period. At each daily collection, the volume of eggs was measured, using a 4‐*l* graduated cylinder, and placed into a separate incubation tank.

Eggs were incubated at 6.1 ± 0.5°C (mean ± *SD*) until they were visually assessed to be at 50% hatch (15.0 ± 0.6 days; mean ± *SD*) at which time genetic samples were taken for 50 individual larvae sampled at random (each whole larva was preserved individually in microtubes containing 250 µl of ThermoFisher RNAlater). During the 94‐day spawning period, daily genetic samples were available for 4,500 larval individuals.

One month after completion of spawning, the 73 adults were sampled, otoliths extracted, and the following morphological traits recorded: total standard length (mm); stomach weight with and without contents (g); total weight (g); liver weight (g); gonad weight (g); and pelvic fin length (mm), a sexually dimorphic trait that appears to affect male cod mating success (Skjæraasen, Rowe, & Hutchings, 2006). The gonadosomatic index (GSI = gonad weight/total body weight) and the hepatosomatic index (HSI = liver weight/total body weight) were calculated as proxies for body condition (Lambert & Dutil, 1997). Due to poor health, two adults were sacrificed early in the spawning season, at which time only length, weight, otolith, and sex were recorded.

Age estimates were obtained from otoliths for 72 of the 73 adults. One otolith from each individual was embedded in a black polyester resin and transversally sectioned at the Otolith Research Laboratory at the Bedford Institute of Oceanography, Canada. Images of sectioned otoliths were then obtained under reflected light, using an Axiocam Mrm camera mounted to a Zeiss SteREO Lumar v12 stereomicroscope. All images were processed to enhance local contrast between the opaque and translucent zones, after which ages were estimated by counting annuli along transects starting from the nucleus in the center of the otolith, proceeding until the edge.

### 2.3 Genetic analysis

Family reconstruction was based on tissue samples from offspring and parents. DNA was extracted from parental fin clips, using an OMEGA Bio‐tek tissue extraction kit, and from whole offspring, using the OMEGA Bio‐tek 96‐well plate DNAeasy extraction kit. All samples were amplified, using two multiplexes consisting of four loci each. Multiplex 1 comprised three tetranucleotide repeat loci (*Gmo8, Gmo19,* and *Tch11*) and one trinucleotide repeat locus (*Gmo35*; Miller et al., 1988; O'Reilly et al., 2002). Multiplex 2 comprised three dinucleotide repeat loci (*Gmo132*,* Gmo2, Tch13*) and one tetranucleotide repeat locus (*Gmo34*; Brooker, 1994; Miller et al., 1988; O'Reilly et al., 2002). Both multiplexes were chosen based on the high levels of heterozygosity at each locus, genotyping reliability, and demonstrated efficiency for paternity studies in Atlantic cod (Dahle, Jørstad, Rusaas, & Otterå, 2006; Wesmajervi, Westgaard, & Delghandi, 2006). Loci were amplified by polymerase chain reaction, as specified by Wesmajervi et al. (2006) and Dahle et al. (2006), and then analyzed using the capillary gel electrophoreses instrument 3130*xl* Genetic Analyzer (Applied Biosystems). Allelic sizes were calculated with instrument‐specific software and the programme GeneMapper (Applied Biosystems). To ensure absolute accuracy in parental genotypes, all adults were amplified three times per multiplex and scored independently by three different people. Disagreements on genotyping identification were referred to a fourth individual. The software MICRO‐CHECKER (van Oosterhout, Hutchinson, Wills, & Shipley, 2004) was used to test the microsatellite loci for evidence of stuttering or null alleles.

Family reconstruction of the allelic data from both offspring and parents was performed with the programme COLONY v2.0.6.1 (Jones & Wang, 2010). Larvae were run in batches of 10 days (~500 larvae per batch). All runs used the full‐likelihood method with high precision and a random seed number. Genotyping error was set to 0.02 per locus. Each analysis was repeated, using medium, long, and very long runs, to assess whether maximum likelihood configuration had been reached. (The length of each run of the programme is determined by the user [Jones & Wang, 2010]; the longer the run, the greater the number of configurations considered in the searching process, and the greater the likelihood that the maximum likelihood configuration will be found.).

### 2.4 Statistical analyses

Individual reproductive success was defined as the number of offspring to which an individual's genotype had been identified as one of those contributing to the fertilization (male) or production (female) of each offspring.

Generalized linear models (GLMs) were used to examine the relative contributions of population identity and trait morphology to individual reproductive success. Models were run separately for each sex, such that the number of offspring produced (reproductive success, or R_S_) was a function of population (inner and outer fjord) and the following morphological variables, measured (with one exception) postmortem: body length (prior to spawning), body weight, HSI, GSI, age, and the residual mean pelvic fin length (calculated from the residuals of linear regressions between pelvic fin length and body length sensu Skjæraasen et al., 2006):

(1) $$R_{S}∼\text{Population}+\text{Length}+\text{Weight}+\text{HSI}+\text{GSI}+\text{Age}+\text{Pelvic}\;\text{Fin}\;\text{Length}$$

Because of the high degree of skewness in the number of sired offspring (see below), the GLM for males incorporated a quasi‐poisson error structure. The model for females was run under the assumption of a normal distribution. Model selection was performed following the protocol suggested by Zuur, Ieno, Walker, Saveliev, and Smith (2009), using stepwise model reduction. Residual plots were examined to ensure appropriate model fits to the data. To examine the robustness of the model selection process and final models, stepwise forward model selection was also performed. All analyses were conducted with R version 3.1.0.

The influence of body size on temporal variation in reproductive success was examined, using linear models run separately for each sex, such that the day of the spawning period was a function of the mean length of fish known to have successfully spawned on each day (as determined by genetic analysis), that is, Day ~Mean length of spawning fish. Mean length was calculated as both the arithmetic and weighted mean lengths of spawning fish (i.e., those whose *R_S_* > 0), the latter being the length of each spawning fish weighted by the relative number of offspring produced by that fish on a given day.

Cumulative rank curves were used to visualize skewness in reproductive success such that the proportion of offspring fertilized was plotted against the rank of the individual in terms of highest number of offspring produced. Deviation from the 1:1 ratio line is indicative of skewed reproductive success. Population differences in reproductive phenotype were examined, using *t* tests.

## 3 RESULTS

### 3.1 Parentage analysis

Microsatellite genotypes were successfully obtained for all putative parents with a minimum of two successful replicate amplifications per locus per adult. There was some evidence of potential null alleles at *Gmo19* (frequency = 0.068) and *Tch11* (0.070) for cod from the inner‐ and outer‐fjord populations, respectively, although it is possible that the MICRO‐CHECKER software misinterpreted minor deviations from Hardy–Weinberg equilibrium (HWE) as evidence of null alleles. Given the lack of consistency of null alleles between populations, and that failure to meet HWE is not typically grounds for discarding a locus (Selkoe and Toonen, 2006), these loci were retained in the analyses. None of the other markers exhibited evidence of scoring error, large allele dropout, or null alleles. Microsatellite genotypes were ultimately obtained for 4,489 of the 4,500 larvae (99.8%). Of the larvae successfully genotyped, 3,508 of 4,489 (78%) comprised all eight loci and 4,459 of 4,489 (99%) comprised four or more loci.

Short and medium runs in COLONY v2.0.6.1 produced variable parentage results whereas results from long and very long runs were nearly identical. Thus, the results from long runs were used for parental assignment. The maximum likelihood was clearly obtained during long runs, providing further indication that the long run provided sufficient time for the programme to reach the best configuration. In the instance where an offspring was assigned to an unknown parent, the genotype of “unknown” parents was compared to the known parental genotypes. If an unknown parental genotype matched at least 5 of 8 loci of a known parent, the unknown parent was reassigned as the known parent. The final parentage analysis resulted in successful paternal assignment to 94.0% of the larvae (4,221 of 4,489) and successful maternal assignment to 93.5% of the larvae (4,198 of 4,489).

### 3.2 Population differences in reproductive success and reproductive phenotype

Reproductive success was significantly influenced by population origin (Table 1). For both males and females, outer‐fjord individuals experienced, on average, lower reproductive success than inner‐fjord females. Comparing males, the average number of offspring fertilized by inner‐fjord males (228.4; range: 0 to 987) was more than three times that of outer‐fjord males (66.1; range: 0 to 351). A threefold difference in reproductive success was also evident among females, inner‐fjord females producing an average 152.5 fertilized eggs (range: 0 to 319) compared to an average of 48.6 eggs (range: 0–266) for outer‐fjord females.

**Table 1:** Output from generalized linear models, after model simplification, between individual reproductive success (number of offspring sired/fertilized) and several fixed effects, all but length having been measured post‐spawning: population identity; length; weight; hepatosomatic index (HSI); gonadosomatic index (GSI); residual mean pelvic fin length

Group	Fixed effect	Slope	*SE*	*t*‐value	*p*‐value
Males	Weight	0.002	0.000	4.752	0.0001
	PopOuter	−1.759	0.419	−4.203	0.0005
	GSI	−0.336	0.160	−2.102	0.0491
Females (all individuals)	Length	1.206	0.390	3.096	0.0040
	Weight	−0.126	0.044	−2.890	0.0070
	PopOuter	−74.496	28.300	−2.632	0.0140
Females (outliers excluded)	Length	0.760	0.300	2.520	0.0180
	PopOuter	−105.830	29.320	−3.610	0.0010
	HSI	−27.010	9.010	−3.000	0.0060

Notes

Data are shown for three groups of spawners: (a) males; (b) females; and (c) a subset of females (for which the two largest individuals were excluded from the analysis). Only significant (*α* < 0.05) fixed effects are presented here; the population fixed effect is represented by “PopOuter”, the outer‐fjord population.

Differences in average reproductive success between populations were not reflected by differences in reproductive phenotype. There were no population differences in average body size at the beginning of the spawning period (mean ± *SD*) for males (inner fjord: 50.9 ± 3.4 cm; outer fjord: 51.7 ± 5.4 cm; *p*‐value = 0.66) or females (inner fjord: 50.9 ± 4.1 cm; outer fjord: 53.5 ± 4.5 cm; *p*‐value = 0.06). Neither post‐spawning condition (males: *p*‐value =0.27, females: *p*‐value = 0.68) nor HSI (males: *p*‐value = 0.11; females: *p*‐value = 0.12) differed between populations. The same was true for average male pelvic fin length (*p*‐value = 0.22). Populations did not differ in the initial day of spawning for males (*p*‐value = 0.864) and females (*p*‐value = 0.114). The only trait that differed between populations was average post‐spawning GSI which was higher among males from the inner fjord (1.2%) compared to those from the outer fjord (0.2%; *p*‐value = 0.014). The same was true for females, the GSI for those from the inner fjord (1.2%) exceeding that for females from the outer fjord (0.7%; *p*‐value < 0.001). As noted previously, neither sex ratio nor number of spawners differed between populations.

### 3.3 Individual differences in reproductive success

**Figure 3:**
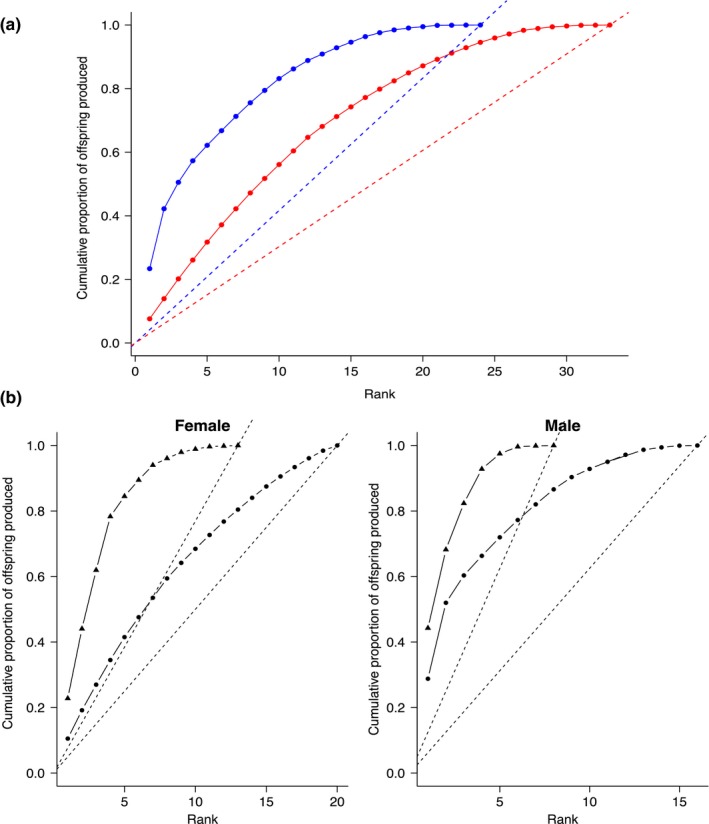
Cumulative proportion of offspring produced by male and female Atlantic cod ranked from most to least successful. Dashed lines represent the relationship if all individuals contributed equally. (a) All data pooled (blue: males; red: females); (b) data separated by population (▲: Outer Fjord; ●: Inner Fjord)

Of the 73 fish in the spawning basin, offspring were assigned to 57 during the spawning period. Overall, males exhibited a greater skew in reproductive success than females (Figure 3). Of the 24 males who contributed gametes, the top‐ranked individual (inner fjord) sired 23.0% of the offspring and the top three males (two from inner fjord, one from outer fjord) were responsible for 50.5% of the fertilized offspring. Females exhibited less of a reproductive skew; among the 33 females who produced fertilized eggs, the top‐ranked female contributed only 7.5% of the offspring. The top three females were responsible for 20.2% of the offspring, substantially less than the male equivalent. When the cumulative reproductive success curves were examined for each population separately, the males and females exhibited skews similar to those evident when the data were pooled (with the exception of the outer‐fjord females, who exhibited a more pronounced skew; Figure 3).

### 3.4 Correlates of reproductive success

Following model simplification, the primary correlates of male reproductive success (number of offspring sired) included body weight, population identity, and GSI (Table 1). Weight was the most significant predictor with a slightly positive coefficient (0.002), indicating that increases in weight had a positive additive effect on the number of offspring sired. As noted above, males from the outer fjord experienced lower reproductive success. The GSI was the least significant predictor, its negative regression coefficient indicating that lower GSI at the end of the spawning period was associated with higher number of offspring sired.

**Figure 4:**
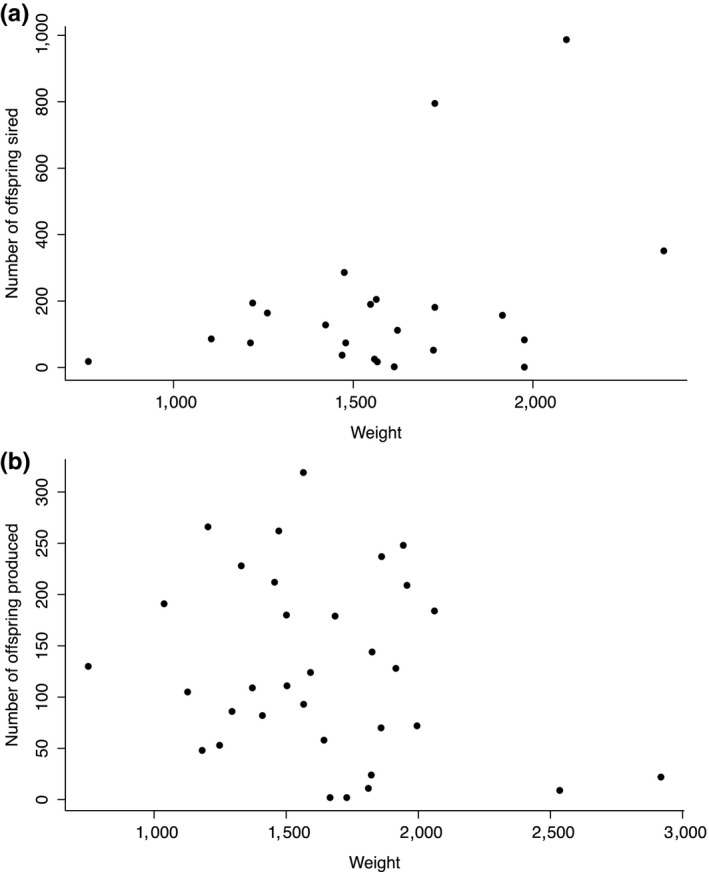
Number of offspring sired as a function of body weight (g) in (a) male and (b) female Atlantic cod (data pooled for both populations)

Regarding body weight, although the regression coefficient was only slightly positive (0.002; Table 1), the statistical significance was high. Upon further examination, it was evident that the three most successful males were among the largest, resulting in a strong positive correlation, given the sample size. However, beyond these three top males, there was little or no relationship between reproductive success and body size (Figure 4). The pattern in these data indicates that there is not a continuous pattern of association between male body size and male reproductive success; if a male was not among the heaviest, weight had no demonstrable effect on reproductive success.

Reproductive success in females was explained by maternal length, population identity, and weight (Table 1). Length had the largest effect, indicative of a positive additive effect on the number of offspring produced. Although the full data set indicated that weight had a negative additive effect on the number of offspring sired, the negative correlation appeared to be heavily influenced by the two largest females both of whom had very low reproductive success (Figure 4b). When these two females were excluded from the analysis, weight was reduced from the model as a non‐significant variable, leaving length, population identity, and HSI as the remaining correlates (Table 1).

### 3.5 Effect of body size on reproductive timing during the spawning period

**Figure 5:**
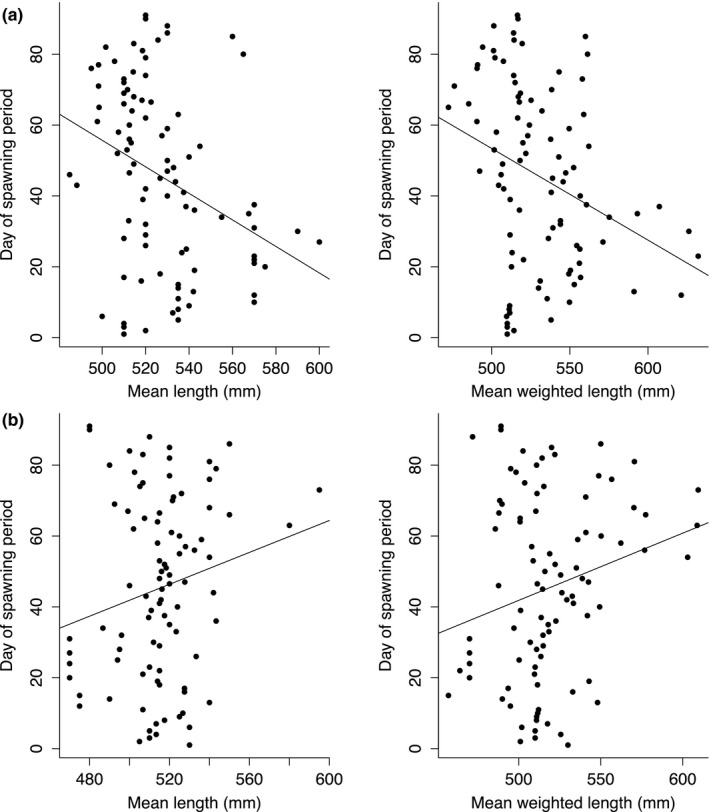
Day of spawning period versus mean length (mm) of reproductively successful (a) male and (b) female Atlantic cod. In the right‐hand plots, mean length is proportionately weighted by the relative number of offspring produced on a particular day. Output from linear models between day of spawning and length: unweighted mean length (males: *p*‐value = 0.001, *r* ^2^ = 0.10; females: *p*‐value = 0.036, *r* ^2^ = 0.04); mean length weighted by the relative number of offspring sired/fertilized on a given day (males: *p*‐value = 0.003, *r* ^2^ = 0.08; females: *p*‐value = 0.076, *r* ^2^ = 0.02)

The relationship between body size and timing of reproduction differed between sexes. Among males, there was a significant negative relationship between day of the spawning period and body size, such that larger males were dominant at the beginning of the spawning period (Figure 5a). Among females, however, larger individuals tended to spawn later than smaller females (Figure 5b). Both models suggest that smaller females were comparatively more active at the beginning of the spawning season. Notwithstanding the statistical significance of most of the associations, the explained variation, as reflected by *r^2^*, was low, being less than or equal to 0.10 for all models. This might be attributable to the observation that individuals of an intermediate size (500–540 mm; both sexes) did not exhibit an obvious temporal pattern in spawning activity (Figure 5).

## 4 DISCUSSION

The present study examined correlates of reproductive success in a broadcast‐spawning marine fish at an exceptionally fine spatial and temporal scale. Based on daily estimates of parentage for almost 4,500 offspring, several broad‐scale patterns emerged. Firstly, despite their small (<10 km) spatial separation, average individual reproductive success differed between the two populations, after accounting for phenotypic variability in several traits. Secondly, reproductive success was skewed within both sexes, albeit much more so among males. Thirdly, body size affected reproductive success differently between sexes, being a strong positive predictor among females but much less so among males. Lastly, body size influenced the timing of reproduction, larger individuals spawning later among females but earlier among males.

The results of the present study indicate that genetically distinctive populations of Atlantic cod (Knutsen et al., 2018, 2011 ) can differ considerably in individual reproductive success when competing for mating and reproductive opportunities. These differences appear not to be attributable to phenotypic variability between populations. Despite similarity in terms of number of individuals, average body size, sex ratio, initiation of spawning period, and body condition, cod originating from outer Risør fjord were less reproductively successful than those from inner Risør fjord, a finding consistent for both males and females. Based on data reported in a separate study (Roney, Oomen, Knutsen, Olsen, & Hutchings, 2018), duration of the spawning period was the same for males but longer for inner‐fjord females.

Our work suggests that there are intrinsic differences between the inner and outer fjord cod populations that affect individual reproductive success. These might be related to population differences in agonistic behavior; males can be significantly more aggressive in some populations than others (Rowe, Hutchings, Skjæraasen, & Bezanson, 2008; aggression contributes to a dominance hierarchy that is associated with fertilization success; Hutchings, Bishop, & McGregor‐Shaw, 1999). Population differences in reproductive success might also be related to differences in sound production by males, mate choice by females, or both (Rowe & Hutchings, 2006). There might also be genetically based differences in reproductive success related to local adaptation, given a high correlation between genetic origin and the presence of three inversion zones in the genome (Sodeland et al., 2016). Cod in the outer fjord are dominated by a North Sea genomic signature, whereas a coastal‐fjord genotype is predominant in inner Risør (Knutsen et al., 2018).

The skew in fertilization probability observed here is well within previously reported estimates of male cod reproductive success. The top three of 28 males in our study fertilized 50% of the total number of eggs produced during the spawning period, an estimate that falls within the range (48%–93%) for the top 3 males (range in number of males: 18–37) reported among four Northwest Atlantic spawning groups (Rowe et al., 2008).

The skew in male reproductive success lends firm support to the existence of a duality of male spawning strategies in cod. The release of gametes by a spawning pair is preceded by a ventral mounting of the female by a single male (Brawn, 1961; Hutchings et al., 1999). Males have also been observed to adopt a satellite strategy and to release milt alongside a spawning pair (Hutchings et al., 1999; Rowe et al., 2008). Studies suggest that males who participate in paired‐spawning events are afforded this opportunity because of their rank within a dominance hierarchy, established by factors such as size and aggressive behavior (Brawn, 1961; Hutchings et al., 1999). Indeed, the most highly successful males in the present study were among the heaviest, lending credence to the hypothesis that they were the top‐ranked males within the dominance hierarchy and, thus, were most likely to participate in paired‐spawning events. For individuals not among the top‐ranked males, body size had little to no effect on reproductive success. Thus, small to moderately heavy males were likely to be lower ranked individuals who, failing to obtain mating opportunities, would be more likely to adopt the satellite male spawning behavior, resulting in lower, but presumably non‐trivial, levels of fertilization success (Rowe et al., 2008).

For females, a skew in reproductive success was evident, although much less so than that observed in males, and the length of a female had a positive effect on the number of offspring produced. The variance in reproductive success among females likely represents variance in a female's fecundity as opposed to differences in mating behavior, given that fecundity is intrinsically linked with female size (Kjesbu, Solemdal, Bratland, & Fonn, 1996; McIntyre & Hutchings, 2003).

An unanticipated finding is that the two largest spawning females had unexpectedly low reproductive success, so much so that they were solely responsible for a negative relationship between weight and the number of offspring in the initial model. Not only were these two females among the three heaviest fish in the spawning basin, the only other fish within their size range was also a female and had zero reproductive success. (Exclusion of these three females from our analyses still yielded highly significant population‐origin effects in the GLM.) The lack of success among the largest females might be attributable to a lack of suitably sized males for paired spawning, possibly due to a physical limitation from males failing to grasp the females during the ventral mount or a behavioral choice on the female's behalf.

The mean size of reproductively successful males decreased over time, suggesting that larger males dominated mating opportunities during the early part of the spawning period. As the spawning season progressed, and larger dominant males presumably began to exhaust their energy and sperm reserves, smaller males were perhaps better able to become more reproductively successful. The temporal shifts in size ranks of reproductively successful males reported here, and elsewhere (Bekkevold, Hansen, & Loeschcke, 2002; Skjæraasen & Hutchings, 2010), for cod might have consequences for the strength and direction of sexual selection in the presence of size‐selective fisheries (Hutchings & Rowe, 2008).

Based on fisheries‐independent survey data, Hutchings and Myers (1993) reported that younger males initiated (and completed) spawning earlier than older males. This might be interpreted as conflicting with our results, although we note that the range in age of cod analyzed by Hutchings and Myers (1993) (6–16 years) was considerably greater than the range considered here (4–8 years). In contrast to males, the mean size of reproductively successful females increased throughout the spawning period, a finding concordant with the experimental work by Hutchings et al. (1999) and the age‐based meta‐analysis by Hutchings and Myers (1993). In contrast, Marteinsdottir and Bjornsson (1999) suggested that larger females begin spawning earlier than smaller females, based on the relative prevalence of females in spawning condition on spawning grounds.

It is has been hypothesized that older, potentially more experienced, individuals achieve higher reproductive success than their younger counterparts (Berkeley, Chapman, & Sogard, 2004; Cardinale & Arrhenius, 2000; Hixon, Johnson, & Sogard, 2013). However, age was not a significant correlate of individual reproductive success in the present study. As noted above, this might be attributable to the limited range in parental age (mean: 5.0 ± 1.0 SD year).

We cannot discount the possibility that the spawning basin might have altered spawning behavior and subsequent levels of reproductive success relative to those that cod would experience under natural conditions. The average depth of the sloped spawning basin (1 m, although deeper in places) was comparatively shallow when compared to the reported depths of many spawning locations in the wild (Rowe & Hutchings, 2003). Environmental variables, such as temperature, were held invariant in the spawning basin; such “constancy” might conceivably have affected behavior. Spawner density was ~2 fish/m^3^, higher than the limited number of estimates of spawner density under natural conditions (0.28–0.31 m^−3^, Morgan, DeBlois, & Rose, 1997; 0.90 m^−3^, Rose, 1993). This might have increased stress among the spawning cod, although only 2.7% of the experimental fish exhibited signs of poor health in our study.

The present study provides novel insights into the spatial scale at which reproductive success can vary within a marine fish species that exhibits high dispersal capabilities. Differential reproductive success between spatially disparate groups of the same species is consistent with the hypothesis that these groups represent different populations and (or) ecotypes at a spatial resolution thought to be uncommon in highly mobile, broadcast‐spawning fish. It is also noteworthy that the inner‐ and outer‐fjord groups of cod examined here are likely to each be predominantly comprised of genomically different cod ecotypes (Knutsen et al., 2018). This raises the intriguing hypothesis that the populations might be diverging because of ecological speciation, that is, the evolution of reproductive isolation between populations resulting from ecologically based but divergent natural selection (Rundle & Nosil, 2005; Schluter, 2000).

Our work contributes to a growing body of research highlighting the influence that the mating system in broadcast‐spawning fish can have on individual reproductive success (Rowe & Hutchings, 2003). Our conclusion of small‐scale population differentiation is also consistent with the finding that the temporal population dynamics of coastal Norwegian cod can be spatially structured, differing among fjords and between sheltered/exposed areas (Rogers, Storvik, Knutsen, Olsen, & Stenseth, 2017). Recent establishment of small‐scale (1 km^2^) marine protected areas provides field‐experimental support for extremely local demographic processes in terms of survival and size structure of coastal Skagerrak cod (Fernández‐Chacón, Moland, Espeland, & Olsen, 2015).

A fundamental challenge to achieving successful resource‐management and conservation outcomes is to correctly identify the spatial scale at which strategies for harvesting and threat mitigation are developed (Cianelli et al., 2010; Conover, Clarke, Munch, & Wagner, 2006; Kuparinen et al., 2016). A mismatch between the spatial scale of a management unit and the spatial scale of a biological unit may result in ineffective actions. Our work suggests that such spatial mismatches exist in marine fishes and that studies of reproductive interactions between putative populations or ecotypes can provide an informative basis on which determination of the scale of adaptation can be ascertained.

## CONFLICT OF INTEREST

None declared.

## AUTHORS’ CONTRIBUTIONS

NER, RAO, and JAH conceived the ideas and designed methodology; NER collected the data with assistance from RAO, HK, and EMO; NER and JAH analyzed the data; NER and JAH led the writing of the manuscript. All authors contributed critically to the drafts and gave final approval for publication.

## DATA ACCESSIBILITY

Data available from the Dryad Digital Repository: https://doi.org/10.5061/dryad.cd000qs.
